# Intraoperative Cardiac Arrest During Anterior Cervical Discectomy and Fusion Due to Takotsubo Cardiomyopathy: A Case Report

**DOI:** 10.7759/cureus.105061

**Published:** 2026-03-11

**Authors:** Vismay Patel, Ariel Litinski, Sujanty Rajaram, Karim Akl

**Affiliations:** 1 Internal Medicine, Hudson Regional Hospital, Bayonne University Hospital, Bayonne, USA; 2 Academy for Biotechnology, Morris County School of Technology, Morristown, USA; 3 Pulmonary and Critical Care Medicine, Hudson Regional Hospital, Bayonne University Hospital, Bayonne, USA

**Keywords:** advanced cardiac life support (acls), anterior cervical discectomy fusion, pulseless electrical activity arrest, takotsubo cardiomyopathy, transthoracic echocardiography (tte)

## Abstract

Intraoperative cardiac arrest is a rare but life-threatening complication of non-cardiac surgery and may result from anesthetic effects, surgical events, or acute cardiopulmonary pathology. We report the case of a 50-year-old man without known structural heart disease who underwent elective C5-C7 anterior cervical discectomy and fusion and developed pulseless cardiac arrest intraoperatively. After approximately 80 minutes of stable anesthesia and completion of the discectomy portion of the operation, end-tidal carbon dioxide abruptly fell to zero and the patient was found to be pulseless. Advanced Cardiac Life Support (ACLS) was initiated for asystole evolving to pulseless electrical activity, with return of spontaneous circulation (ROSC) followed by a second arrest requiring repeat resuscitation and subsequent sustained ROSC. Point-of-care echocardiography demonstrated severe new left ventricular systolic dysfunction with apical akinesis and basal hyperkinesis. Cardiac biomarkers were elevated. Emergent coronary angiography demonstrated no obstructive coronary artery disease, and left ventriculography revealed apical ballooning consistent with Takotsubo cardiomyopathy. The patient required temporary vasoactive and inotropic support, improved hemodynamically within 48 hours, and demonstrated complete recovery of left ventricular systolic function by postoperative day 5 with full neurologic recovery. This case highlights Takotsubo cardiomyopathy as an important consideration in unexplained intraoperative cardiovascular collapse.

## Introduction

The perioperative period is associated with significant physiologic and emotional stress, even in patients considered low risk. Exposure to general anesthesia, surgical stimulation, and sympathetic activation increases the risk of cardiopulmonary complications, including arrhythmias, myocardial ischemia, cardiogenic shock, and, rarely, cardiac arrest [1]. Determining the underlying etiology of intraoperative cardiac arrest is inherently challenging, as patients cannot report symptoms and early management appropriately prioritizes resuscitation.

Takotsubo cardiomyopathy - also referred to as stress cardiomyopathy - is an uncommon but increasingly recognized cause of acute, reversible left ventricular systolic dysfunction. Although it frequently mimics acute coronary syndrome, Takotsubo cardiomyopathy can rarely present with malignant arrhythmias or cardiac arrest [2]. First described following acute emotional stress [3], it has since been associated with physical stressors, neurologic disease, medications, and perioperative events associated with catecholamine excess [4,5]. Awareness of this entity in the operating room is essential, as early recognition may guide diagnostic evaluation and management.

Takotsubo cardiomyopathy accounts for approximately 1-2% of patients presenting with suspected acute coronary syndrome and most commonly occurs in postmenopausal women, although it can also occur in men and in perioperative settings involving significant physiologic stress. Intraoperative presentations are uncommon but may manifest as sudden hemodynamic collapse, malignant arrhythmias, cardiogenic shock, or cardiac arrest [2]. Recognizing perioperative triggers and early warning signs, including unexplained hypotension or new ventricular dysfunction in the absence of coronary obstruction, is important for early diagnosis and management.

## Case presentation

A 50-year-old man with cervical myelopathy presented for elective anterior cervical discectomy and fusion at C5-C7 after failure of conservative management. Ten days prior to surgery, he underwent outpatient cardiology evaluation for preoperative risk stratification and was deemed to have no cardiac contraindication to surgery. Transthoracic echocardiography demonstrated a normal left ventricular ejection fraction (LVEF) of 60%.

His medical history included hypertension, hyperlipidemia, well-controlled type 2 diabetes mellitus (HbA1c 6.6%), and gastroesophageal reflux disease. He had undergone prior spine surgery approximately one year earlier without anesthetic complications. Home medications included lisinopril, metformin, baclofen, gabapentin, and atorvastatin. He denied tobacco, alcohol, or illicit drug use. Preoperative laboratory studies, including complete blood count, comprehensive metabolic panel, and urine drug screen, were within normal limits. He was classified as American Society of Anesthesiologists (ASA) physical status II.

General anesthesia was induced with fentanyl 100 mcg, lidocaine 100 mg, propofol 200 mg, and succinylcholine 120 mg intravenously, followed by uncomplicated endotracheal intubation. Anesthesia was maintained with a propofol infusion at 250 mcg/kg/min. Mechanical ventilation was initiated in synchronized intermittent mandatory ventilation mode with FiO₂ 100%, respiratory rate 14 breaths/min, and tidal volume 600 mL. The patient remained normothermic, and end-tidal CO₂ ranged from 39 to 44 mmHg. Hemodynamics were stable for approximately 80 minutes. No preceding tachycardia, hypertension, or progressive bradycardia was documented prior to the event. The first abnormal intraoperative finding was an abrupt decrease in end-tidal carbon dioxide to zero, which prompted immediate pulse assessment and recognition of cardiac arrest.

The anterior cervical discectomy was completed without significant blood loss. According to the operative report, the surgical field was stable at the time of the cardiovascular collapse, and no significant manipulation of the carotid sheath or retractor repositioning was documented immediately preceding the arrest. Before the surgical team could proceed with the fusion portion of the operation, end-tidal CO₂ abruptly decreased to 0 mmHg. A carotid pulse was not palpable, and Advanced Cardiac Life Support (ACLS) was initiated. The initial rhythm was asystole, later evolving into pulseless electrical activity. Return of spontaneous circulation (ROSC) was achieved; however, the patient experienced a recurrent arrest requiring a second round of ACLS, after which sustained ROSC was obtained. The surgical wound was dressed, a hard cervical collar was placed, and the patient was transferred to the intensive care unit (ICU) on vasoactive support.

Immediately following resuscitation, point-of-care ultrasonography demonstrated severely reduced left ventricular systolic function with akinetic apical segments and hyperkinetic basal segments. Right ventricular size and systolic function were preserved, and no pericardial effusion was present. These findings narrowed the differential diagnosis by making massive pulmonary embolism, cardiac tamponade, and tension pneumothorax less likely.

Prior to ICU transfer, the patient received approximately 3 liters of normal saline bolus and was supported with norepinephrine titrated up to 30 mcg/min, with systolic blood pressure in the low 100s. In the ICU, milrinone (0.375 mcg/kg/min) and vasopressin (0.03 units/min) were initiated for cardiogenic shock physiology and hemodynamic support. Laboratory testing revealed elevated cardiac troponin levels consistent with myocardial injury. Although troponin elevation may occur following cardiac arrest due to global myocardial ischemia during resuscitation, further evaluation was performed to exclude acute coronary syndrome.

**Figure 1:**
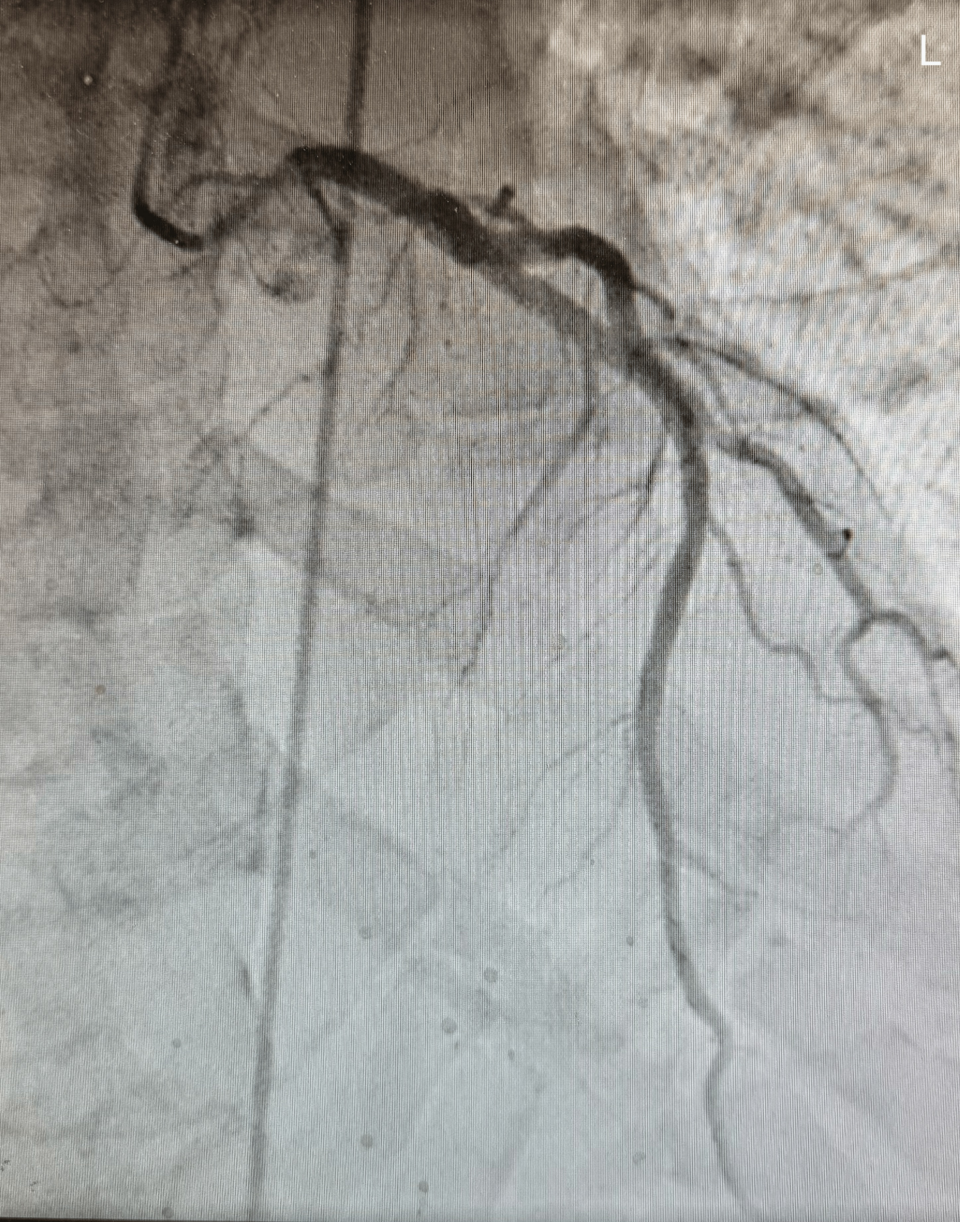
Patent left coronary

**Figure 2:**
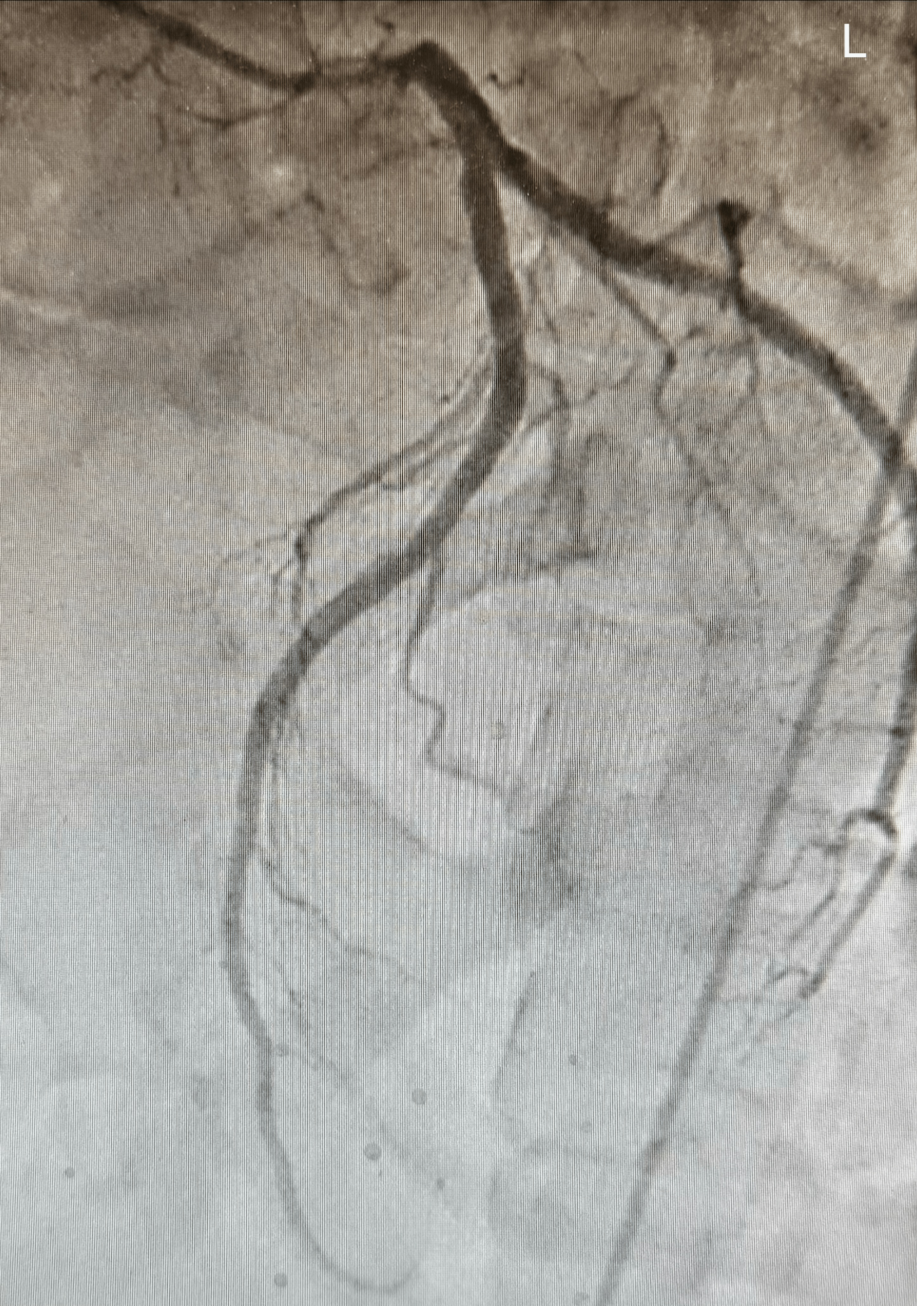
Left coronary artery

**Figure 3:**
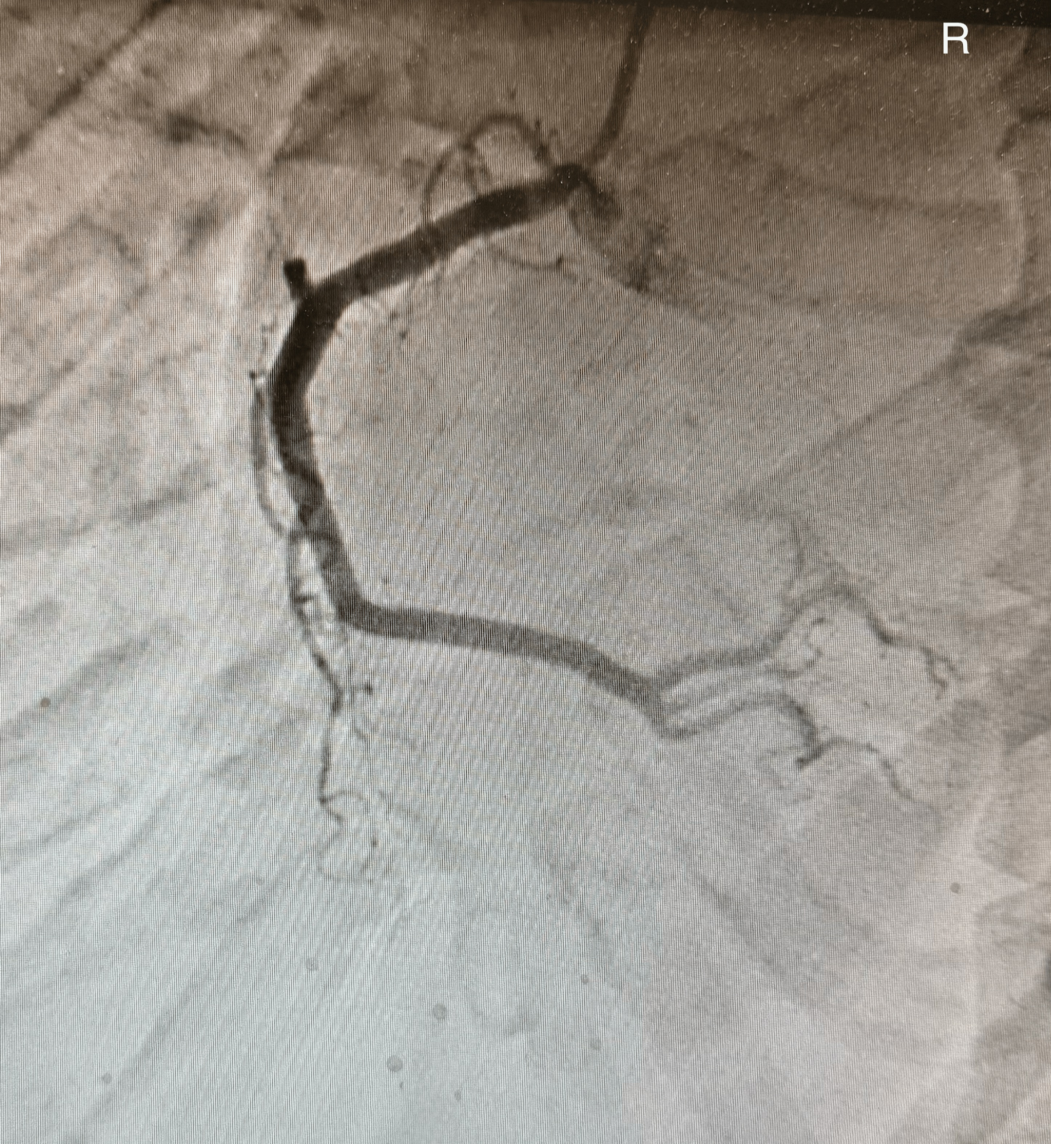
Right coronary artery

Emergent cardiology consultation was obtained, and the patient underwent urgent coronary angiography and left ventriculography. Coronary angiography demonstrated no clinically significant obstructive coronary artery disease (Figures 1-3).

Coronary angiography of the left and right coronary system demonstrated preserved flow without evidence of significant obstructive coronary artery disease, supporting a non-ischemic etiology for the patient’s acute left ventricular systolic dysfunction.

**Figure 4:**
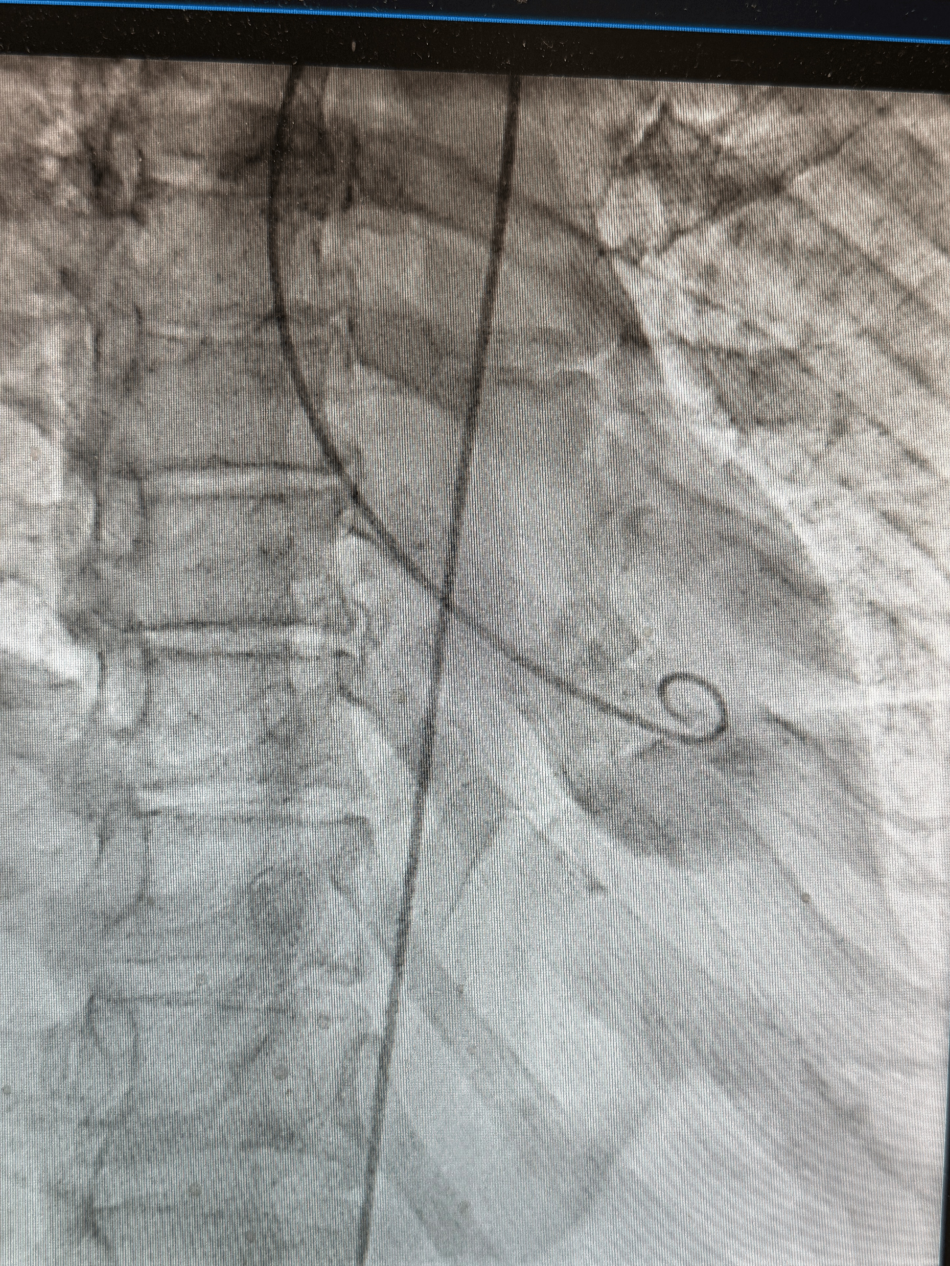
Left ventriculography demonstrating apical ballooning. End-systolic ventriculographic image demonstrating apical akinesis with relative hypercontractility of the basal segments, producing the characteristic apical ballooning morphology consistent with Takotsubo cardiomyopathy.

Left ventriculography demonstrated severely depressed left ventricular systolic function with apical ballooning and relative hyperkinesis of basal segments, a characteristic imaging pattern consistent with Takotsubo cardiomyopathy (Figure 4, Video 1).

Left ventriculography demonstrated apical akinesis with basal hyperkinesis during systole, consistent with stress-induced (Takotsubo) cardiomyopathy (Video 1).

**Video 1:** Apical ballooning

Left ventricular end-diastolic pressure was 12-15 mmHg. Hemodynamics improved over the following days, and norepinephrine and vasopressin were discontinued by postoperative day 2. Repeat transthoracic echocardiography on postoperative day 5 demonstrated normalization of LVEF. Neurologic evaluation, including computed tomography of the head and electroencephalography, showed no acute intracranial pathology or seizure activity. On postoperative day 6, the patient returned to the operating room to complete the anterior cervical discectomy and fusion (C5-C7), which proceeded without complication. He was extubated the following day, milrinone was discontinued, and he recovered full cognitive function. The remainder of hospitalization was unremarkable, and he was discharged home three days later.

Post-resuscitation transthoracic echocardiography demonstrated marked global left ventricular hypokinesis with severely reduced systolic function and no significant valvular abnormalities or pericardial effusion. Follow-up echocardiography performed during the Takotsubo evaluation demonstrated complete recovery of left ventricular systolic function, with an estimated ejection fraction of 55-60%, normal global and segmental wall motion, preserved right ventricular function, and no significant valvular pathology (Video 2).

**Video 2:** Echocardiography showing normal LV function

Takotsubo cardiomyopathy is characterized by acute, profound left ventricular systolic dysfunction with subsequent full recovery, often following severe physiologic or emotional stress.

## Discussion

Although Takotsubo cardiomyopathy most commonly affects postmenopausal women, cases occurring in men and perioperative settings have been increasingly reported in association with significant physiologic stress. Takotsubo cardiomyopathy is an acute and generally reversible syndrome characterized by transient left ventricular systolic dysfunction and regional wall-motion abnormalities, typically triggered by emotional or physical stress and associated with catecholamine excess [2,3,6-10]. It accounts for approximately 1-2% of patients presenting with suspected acute coronary syndrome and elevated cardiac biomarkers. Although overall prognosis is often favorable, perioperative Takotsubo cardiomyopathy has been associated with more severe presentations, including cardiogenic shock and cardiac arrest.

The pathophysiology remains incompletely understood. Leading mechanisms include catecholamine-mediated myocardial stunning and myocardial microvascular dysfunction [6-8]. Transient coronary vasospasm and endothelial dysfunction may also contribute [9,10]. Diabetes mellitus is associated with endothelial and microvascular dysfunction and may increase susceptibility to microcirculatory dysregulation during acute perioperative stress.

The precise trigger for Takotsubo cardiomyopathy in this case remains uncertain; however, perioperative physiologic stress and autonomic imbalance may contribute to catecholamine-mediated myocardial stunning. Excess sympathetic activation and circulating catecholamines are believed to play a central role in the development of transient ventricular dysfunction observed in stress cardiomyopathy [6].

Intraoperative cardiac arrest carries a broad differential diagnosis, including anesthetic complications, myocardial infarction, pulmonary embolism, tamponade, tension pneumothorax, hemorrhage, and metabolic derangements. Definitive diagnosis requires evidence of myocardial injury and exclusion of other causes of acute left ventricular dysfunction, particularly obstructive coronary artery disease, acute myocarditis, and pheochromocytoma [11-13]. In this case, point-of-care echocardiography proved invaluable in rapidly narrowing the differential diagnosis. Point-of-care echocardiography is particularly useful in rapidly identifying ventricular dysfunction, right ventricular strain, or pericardial effusion during perioperative cardiovascular collapse [14]. Preserved right ventricular function and absence of pericardial effusion reduced suspicion for massive pulmonary embolism and tamponade, while the pattern of apical akinesis with basal hyperkinesis supported stress cardiomyopathy.

Cervical spine procedures may rarely trigger vagally mediated reflexes resulting in bradycardia or transient asystole due to carotid sinus stimulation or traction on autonomic structures. Although this mechanism was considered in the differential diagnosis, the absence of progressive bradycardia preceding the event and the presence of severe new left ventricular systolic dysfunction with characteristic apical ballooning supported Takotsubo cardiomyopathy as the more likely etiology.

Management is primarily supportive, focusing on hemodynamic stabilization until myocardial recovery occurs. Takotsubo cardiomyopathy accounts for approximately 1-2% of patients presenting with suspected acute coronary syndrome and most commonly affects postmenopausal women, although cases have also been reported in men and perioperative settings [15,16]. For patients who experience perioperative Takotsubo cardiomyopathy, careful evaluation prior to future surgical procedures is recommended. Strategies may include close perioperative monitoring, minimizing excessive sympathetic stimulation, and early use of echocardiography in cases of unexplained hemodynamic instability. Although recurrence is uncommon, awareness of this condition may help guide perioperative planning and risk mitigation [17]. Recovery of ventricular function typically occurs within days to weeks [17,18]. Despite its reversible nature, Takotsubo cardiomyopathy may be associated with significant complications, including malignant arrhythmias and recurrent cardiac arrest [19].

Takotsubo cardiomyopathy should be considered in cases of unexplained intraoperative cardiac arrest or cardiogenic shock, even in patients without known structural heart disease. Point-of-care echocardiography is a critical diagnostic tool during perioperative cardiovascular collapse, allowing rapid differentiation between ischemic and non-ischemic causes. Early coronary angiography and ventriculography are essential to exclude obstructive coronary disease and confirm Takotsubo cardiomyopathy, guiding appropriate supportive management.

## Conclusions

Takotsubo cardiomyopathy is an uncommon but important cause of unexplained intraoperative cardiovascular collapse. Clinicians should consider this diagnosis when sudden cardiac arrest or cardiogenic shock occurs during non-cardiac surgery without an obvious etiology. Early use of point-of-care echocardiography and prompt coronary angiography can facilitate accurate diagnosis and guide appropriate supportive management in this reversible condition.

## References

[1] Farooq Z, Malik S, Bhat M, Farooq S (2025). Perioperative cardiac complications and evidence-based strategies for their management. Cureus.

[2] Arbel Y, Ben-Assa E, Puzhevsky D (2019). Forced diuresis with matched hydration during transcatheter aortic valve implantation for Reducing Acute Kidney Injury: a randomized, sham-controlled study (REDUCE-AKI). Eur Heart J.

[3] Kurisu S, Sato H, Kawagoe T (2002). Tako-tsubo-like left ventricular dysfunction with ST-segment elevation: a novel cardiac syndrome mimicking acute myocardial infarction. Am Heart J.

[4] Santoro F, Núñez Gil IJ, Arcari L (2024). Neurological disorders in Takotsubo syndrome: clinical phenotypes and outcomes. J Am Heart Assoc.

[5] Arunkumar S, Jegaverrapandi K (2024). Pharmacological triggers of Takotsubo cardiomyopathy: an updated review of evidence and recommendations. Curr Cardiol Rev.

[6] Wright PT, Tranter MH, Morley-Smith AC, Lyon AR (2014). Pathophysiology of Takotsubo syndrome: temporal phases of cardiovascular responses to extreme stress. Circ J.

[7] (2016). What's new in the European Society of Cardiology 2016 Guidelines for the diagnosis and treatment of acute and chronic heart failure?. Eur Heart J.

[8] Sandhu K, Nadar SK (2015). Percutaneous coronary intervention in the elderly. Int J Cardiol.

[9] Bechara LR, Moreira JB, Jannig PR (2014). NADPH oxidase hyperactivity induces plantaris atrophy in heart failure rats. Int J Cardiol.

[10] Hung MJ, Ko T, Liang CY, Kao YC (2017). Two-dimensional myocardial deformation in coronary vasospasm-related Takotsubo cardiomyopathy: a case report of a serial echocardiographic study. Medicine (Baltimore).

[11] Prasad A, Lerman A, Rihal CS (2008). Apical ballooning syndrome (Tako-Tsubo or stress cardiomyopathy): a mimic of acute myocardial infarction. Am Heart J.

[12] Ghadri JR, Wittstein IS, Prasad A (2018). International expert consensus document on Takotsubo syndrome (Part I): clinical characteristics, diagnostic criteria, and pathophysiology. Eur Heart J.

[13] Eitel I, von Knobelsdorff-Brenkenhoff F, Bernhardt P (2011). Clinical characteristics and cardiovascular magnetic resonance findings in stress (Takotsubo) cardiomyopathy. JAMA.

[14] Brinkman S, de Keizer NF, de Lange DW, Dongelmans DA, Termorshuizen F, van Bussel BC (2024). Strain on scarce intensive care beds drives reduced patient volumes, patient selection, and worse outcome: a national cohort study. Crit Care Med.

[15] Hessel EA (2021). Perioperative Takotsubo syndrome. Can J Anaesth.

[16] Deshmukh A, Kumar G, Pant S, Rihal C, Murugiah K, Mehta JL (2012). Prevalence of Takotsubo cardiomyopathy in the United States. Am Heart J.

[17] Yerasi C, Koifman E, Weissman G (2017). Outcomes of stress-induced cardiomyopathy. Eur Heart J Acute Cardiovasc Care.

[18] Singh K, Carson K, Shah R (2014). Meta-analysis of clinical correlates of acute mortality in Takotsubo cardiomyopathy. Am J Cardiol.

[19] Templin C, Ghadri JR, Diekmann J (2015). Clinical features and outcomes of Takotsubo (stress) cardiomyopathy. N Engl J Med.

